# Using a Smartphone Application to Promote Healthy Dietary Behaviours and Local Food Consumption

**DOI:** 10.1155/2015/841368

**Published:** 2015-08-25

**Authors:** Jason Gilliland, Richard Sadler, Andrew Clark, Colleen O'Connor, Malgorzata Milczarek, Sean Doherty

**Affiliations:** ^1^Department of Geography, School of Health Studies, Department of Paediatrics Social Science Centre, The University of Western Ontario, 1151 Richmond Street, London, ON, Canada N6A 5C2; ^2^Department of Family Medicine, Michigan State University, 200 E 1st Street, Flint, MI 48502, USA; ^3^Department of Geography, Social Science Centre, The University of Western Ontario, 1151 Richmond Street, London, ON, Canada N6A 5C2; ^4^Division of Food and Nutritional Sciences, Brescia University College, 1285 Western Road, London, ON, Canada N6G 1H2; ^5^Department of Geography, Wilfrid Laurier University, 75 University AW, Waterloo, ON, Canada N2L 3C5

## Abstract

Smartphone “apps” are a powerful tool for public health promotion, but unidimensional interventions have been ineffective at sustaining behavioural change. Various logistical issues exist in successful app development for health intervention programs and for sustaining behavioural change. This study reports on a smartphone application and messaging service, called “SmartAPPetite,” which uses validated behaviour change techniques and a behavioural economic approach to “nudge” users into healthy dietary behaviours. To help gauge participation in and influence of the program, data were collected using an upfront food survey, message uptake tracking, experience sampling interviews, and a follow-up survey. Logistical and content-based issues in the deployment of the messaging service were subsequently addressed to strengthen the effectiveness of the app in changing dietary behaviours. Challenges included creating relevant food goal categories for participants, providing messaging appropriate to self-reported food literacy and ensuring continued participation in the program. SmartAPPetite was effective at creating a sense of improved awareness and consumption of healthy foods, as well as drawing people to local food vendors with greater frequency. This work serves as a storehouse of methods and best practices for multidimensional local food-based smartphone interventions aimed at improving the “triple bottom line” of health, economy, and environment.

## 1. Background

The production and consumption of healthy, local food have numerous environmental, economic, and public health benefits. Unfortunately, many people experience or perceive barriers to accessing such foods. Access to healthy food is of increasing interest to public health researchers and practitioners as research suggests links between the level of accessibility to (un)healthy food and the prevalence of obesity, type 2 diabetes, and other diet-related diseases [1–3]. The recent evolution of food retailing practices has contributed to geographic gaps in access to healthy foods, a phenomenon commonly known as “food deserts” [4–7]. Prolonged exposure to food deserts can contribute to inequalities in health outcomes [8, 9], even where individuals can physically access healthy foods; however, additional economic, educational, and behavioural constraints can limit real opportunities for behavioural change [10, 11].

This paper presents results for the preliminary phase of the “SmartAPPetite” research project: a smartphone application, or “app,” designed to encourage healthy eating by reducing educational, behavioural, and economic barriers to accessing healthy, local food. (In this study, local food refers to foods that are either grown or have value added (e.g., processed, fermented, ground) within the economic region of Southwestern Ontario.) SmartAPPetite uses a direct “push notification” method to deliver specialized food messaging (nutrition and healthy eating tips, recipes, and local food vendor information) via smartphones to help participants reach their food-related goals and help local food vendors increase sales. The theoretical framework discussed below provides justification for the research objectives and methodology, and is grounded in using a behavioural economics approach to behaviour change. Furthermore, discussion of gaps in the literature supports the theoretical and empirical contributions discussed later in the paper.

### 1.1. Theoretical Framework

Many programs addressing diet-related health inequalities have centered on structural change to the food system (e.g., through a new food retail source) [12], but a common behavioural approach has been to increase awareness of the importance of healthy eating through educational programs [13, 14]. Unfortunately, educational programs can be of limited utility due to behavioural factors, because knowledge of healthy eating habits does not always translate into practice [15, 16]. Any behavioural approach must consider education and behavioural cues. The distinction between education and behaviour is clear when considering the difference between classical and behavioural economics [17], while classical economics assumes rational and optimal decision-making (and thus, education implies behaviour), behavioural economics concedes that humans commit predictably irrational decisions which compromise their optimal health and well-being [18, 19].

The essence of a behavioural economic approach is “to use decision errors that ordinarily hurt people to instead help them” (page 2) [20]; for instance, by capitalizing on the status quo bias and making the better (or healthier) option the default choice [21]. Thaler and Sunstein [22] showed that behavioural access can be improved by creating incentives for healthy eating through product placement and suggestive advertising.

The technique of incentivizing healthy choices is commonly referred to as “nudging,” or libertarian paternalism, because unhealthy choices are not taken away from the choice environment. Rather, healthier choices are simply made the default choice by reframing the architecture of various levels of the food environment. Generally, recognizing the difference between educational and behavioural factors will lead to more relevant policy and program development. This theoretical framework inspired the SmartAPPetite project, which aims to make use of an everyday technology, smartphones, to influence health behaviour change.

### 1.2. Smartphones and Health Promotion Apps

Smartphones present an excellent opportunity to advance the work of behavioural economics theory because of the sheer volume of users, 56% of adults, and the frequency with which people use this technology (and thus, the opportunity to reshape consumer habits by making healthy decisions “easy” through a commonly used product) [23]. This ubiquity provides a major opportunity to influence behavior, typically at a lower cost of implementation compared to other technologies [24, 25]. To understand the significance of smartphone apps for encouraging consumption of healthy or local food, however, an appropriate research design grounded in behavioural economics must be implemented.

In the social science environment, natural experiments have been advocated by researchers as a useful tool for demonstrating causality of diet-related health outcomes [2, 26, 27]. App development presents an opportunity to institute direct, controlled experiments on users and nonusers of smartphones. But evaluation should necessarily cover a range of methods as indicated by Schäfer Elinder and Jansson [27]: “findings in quantitative studies need to be verified through qualitative research exploring people's own views and experiences on their opportunities and barriers to a healthy lifestyle” (page 312). Within a behavioural economic framework, this mixed-methods approach yields not only objective measures of behaviour change but also reasons as to why users felt the intervention was effective at changing behaviour.

A literature review of studies which utilized or evaluated smartphone interventions for behaviour change yielded 53 research papers and 6 systematic review papers [28–33] (a full list of references is available from the authors upon request). Most studies used experimental study designs to isolate the impact of a smartphone app or messaging service. The studies addressed a wide range of health concerns in their messaging, including diet, physical activity, obesity/weight, diabetes, cardiovascular disease, alcohol/smoking cessation, and sexual and mental health.

Of the 53 research papers, only 9 addressed weight or BMI [34–42], and just 6 directly addressed issues of diet in their study designs [37, 43–47]. 21 of 26 studies which considered healthy behaviours reported a positive effect of their program. Of the 6 papers which measured effects on dietary behaviours, 4 reported positive effects [43, 44, 46, 48]. Of the 18 studies that reported on impacting knowledge and awareness of healthy behaviours, only one did not find a positive impact [49]. While these results provide a strong rationale to pursue a behaviour change intervention focused on food literacy and healthy food consumption, most of these studies focused only on single-tiered interventions.

### 1.3. Addressing a Gap in the Literature

Despite the overall positive results reported in the intervention literature, the long-term effects of unidimensional programs have been questioned. Algazy et al. [50] reported that “single-intervention programs, such as low-calorie diets and exercise regimens, generally produce only modest weight loss” (page 7). As well, long-term behavioural change can be difficult to demonstrate in the absence of follow-up programs [51]. Although some studies have shown evidence of short-term effects regarding a behavioural outcome, few were able to demonstrate this in the long-term [31]. Messaging which provides advice on specific healthy behaviours to the exclusion of other considerations can lack effectiveness or even act against positive behavioural change. Johnson et al. [52] suggest that “providing information about a particular issue…can have unintended consequences such as reducing attention about important issues…or increasing focus on only a single corrective action” (page 499). For instance, by overemphasizing calorie counting, a messaging program could inadvertently increase sodium intake as participants seek out low-calorie foods to the exclusion of other nutritional qualities. This speaks to the effect of marketing and the importance of recognizing behavioural economic principles [18, 19]. Such warnings also demonstrate a need to devise interventions which address multiple layers of nutritional information and approaches to effecting behaviour change.

Gittelsohn and Lee [53] argued that “a mixed educational-environmental-behavioural economic approach will work because it addresses different components of individual (and group) decision-making. Decisions should be informed (educational), constrained (environmental), and guided (behavioural)” (page 60). Supporting this assertion, one review notes that technology interventions (text messaging or smartphone applications) supported either by education or an additional intervention demonstrated a beneficial impact by reducing physical inactivity and/or overweight/obesity [30].

None of the evaluated articles addressed a multidimensional issue with a multidimensional intervention approach. But food especially has a critical connection to economic and environmental concerns which, when implemented into a healthy eating intervention, could yield positive results across the “triple bottom line”: economy, environment, and health [54]. Gittelsohn and Lee [53] offer important motivation for a multidimensional intervention which engages multiple aspects of the food system: “often the healthy eating discussion focuses on the retailer-consumer food system, but engagement with retailers, distributors, producers, and manufacturers could also greatly influence dietary outcomes” (page 64). Designing a behaviour change tool which addresses more than just a health issue, such as obesity, through a multidimensional approach is thus both empirically novel and a significant contribution to the literature on health promotion and the theory on behavioural economics.

### 1.4. Research Objectives

The first objective was to develop, test, and improve the functionality and user-friendliness of a smartphone app intervention tool (SmartAPPetite) for improving the knowledge, purchasing, and consumption of healthy, local food. Simultaneously, the second objective was to gather essential data on participant demographics to help tailor the program to the desires of participants. The final objective entailed implementing the intervention and assessing perceived and actual changes in food literacy, purchasing, consumption, and self-rated health to determine the impacts of the multitiered program on participants.

## 2. Methods/Design

### 2.1. Study Design

SmartAPPetite's design was guided by Atkins and Michie's [55] principles of individual behaviour change (capability, opportunity, motivation), which implicitly recognize the need to overcome the competing, suboptimal choices of central concern in behavioural economics. SmartAPPetite was designed to address the concern that “health promotion approaches to changing behaviour have focused on giving information and largely ignored the role of motivational, social, and environmental factors” (page 31) [55] which predispose people to making such suboptimal choices. Building on previous studies which evaluated single-dimensional or self-directed food messaging [34, 41], SmartAPPetite used direct researcher engagement and multidimensional “info chains” of healthy eating tips, recipes, and vendor spotlights/coupons to “nudge” participants from personally-defined food goals directly to making healthy purchases at local food vendors. These message chains are hypothesized to be more effective because they provide different types of linked information desired by consumers and reinforce healthy behaviours through persistent messaging. Given issues of inferring causality in quasi-experimental study designs, direct causal inferences cannot be drawn; rather, the goal is to add to the knowledge on smartphone apps for behaviour change.

The application development phase also drew on Hebden et al.'s [56] development process for ensuring successful app creation. The current study likewise used an iterative development process marked by: involvement from potential participants and professionals from fields in marketing, nutrition, and information technology; an exploration of behaviour change strategies; and several tests of prototype apps [56]. Given the ever-present nature of new technologies such as smartphone apps and the recent growth in the popularity of local food networks, great potential exists to change healthy eating and local food behaviours through this app.

### 2.2. Message Development Strategy

The most critical study design element, and the first key step in Hebden et al.'s process [56], was to devise appropriate food messaging that would be both instructional and encouraging to participants. The messaging needed to generally educate participants about the nutritional and economic value of local food, but also help them reach their own diet-related goals. As relevant, many of Abraham and Michie's [57] 26 validated behaviour change techniques were used to ensure message creation and deployment were adequately informational and encouraging, including: providing information about the behaviour-health link, consequences, and contingent rewards; prompting intention formation, instruction, and specific goal setting; and using follow-up prompts, motivational interviewing, and time management tips.

Prior to the implementation of the intervention, the research team's registered dietitian and project manager worked closely with research assistants with backgrounds in nutrition and health promotion to devise food messaging tips. Messaging was developed from available and credible nutritional advice on Canadian dietitian and public health websites to reflect various levels of food literacy. Messages were then linked to recipes which included relevant food items to encourage participants to act on this food knowledge.

The process of sending a message had two components. First, SmartAPPetite drew from and assigned a unique URL link to a list of sub-160 character messages (abbreviated using Google URL shortener). For example, “potassium, magnesium, and calcium work together to lower blood pressure. Do you know how much you should consume daily? Click here.” Links provided participants with further information about the health tip and, if included, the vendor featured in the message. A web analytics program was used to discern whether participants followed these links forward to other websites (as discussed below). Second, the team worked with local food vendors to procure discounts and create vendor “spotlights,” whereby participants would receive messaging about featured healthy foods and food products when near a relevant food vendor.

The impetus for these info chains comes from behavioural economics, with the idea that creating new, healthy food-oriented heuristics in an individual's food environment, and especially by using a technology so common to their lifestyle, can help nudge them into making healthy choices [58]. Ultimately, 95 unique food info chains (out of 309 created by the team) were sent to users over a 10-week study period.

### 2.3. Recruitment

The study took place at the Western Fair Farmers' and Artisan's Market in London, Ontario, Canada (see http://www.londonsfarmersmarket.ca/; [59]). Recruitment was conducted actively and passively at the market by pairs of research assistants. Patrons were verbally informed about the study and, if interested in participating, were provided a printed letter of information and letter of consent to participation. After signing the letter of consent, participants were registered in the study via a project website and instantly began receiving messages.

Over the course of two Saturday market days, the team recruited 208 participants who represented a range of market visitors and community members. The market attracts between 2000 and 2500 visitors weekly; thus, this represents about a 10% sample of all market-goers [59]. Throughout the study, participation was incentivized through vendor coupons and gift card draws for participants.

### 2.4. Data Collection

To achieve the ultimate goal of creating a self-sustaining healthy eating/local food smartphone app, the team needed to understand more about the food related goals and behaviours of study participants, and how these may vary by sociodemographic characteristics. The team used mixed methods for collecting data, including: (1) an* upfront food survey* to assess dietary habits and goals before receiving the intervention; (2)* message uptake tracking* online using Google Analytics (GA); (3)* experience sampling* during the intervention through telephone interviews; and (4) a* follow-up food survey* to assess change in dietary habits and goals after the intervention.

The upfront survey included questions pertaining to household demographics, allergens/restrictions, diet and health-related goals, and food purchasing and consumption habits. Baseline purchasing and consumption were measured by participants indicating how many times per week they currently consume/purchase a list of common food items, as well as where products were purchased. Participants were then placed into “bins” based on various dietary restrictions and diet-related goals to enable individually-tailored food messaging.

The intervention period lasted between 8 and 10 weeks for each participant, during which time they received 2 to 3 daily messages about healthy eating, healthy recipes, and information about local food vendors at the market. As well, participants had the option to “check-in” at the market on Saturdays to obtain day-specific deals at participating healthy food vendors.

The second method of data collection entailed* online tracking of message uptake*. Because each message included a unique URL that users could click for further details, the GA web interface was used to track the frequency of URL page views, exit rates from the site, visit durations, and other factors indicative of information utility [60].

During the intervention period, participants were contacted for a* short interview* on their personal experience with SmartAPPetite. The intent was to capture their experience to date and make suggestions to improve and customize their experience for the remainder of the study (e.g., changes in message type, frequency, or delivery time). Questions focused on the utility of the messages/information; any changes in purchasing habits, food preparation, and/or consumption; and how SmartAPPetite could be improved.

After the intervention, the team administered a* follow-up survey* combining questions from the upfront and experience sampling surveys, which enabled consideration of SmartAPPetite's effect on the purchasing and/or consumption of healthy, local foods, along with the participant's overall experience.

## 3. Results

Most critically, this study found that participants who were more engaged with SmartAPPetite experienced more positive changes in healthy food consumption. The specific results reported here provide a broad lens for determining successful elements of the SmartAPPetite application and future adjustments necessary to improve its effectiveness.

From a total of 208 participants in the intervention, the team collected 207 upfront surveys (99.5%), 123 experience sampling phone interviews (59.1%), 123 follow-up surveys (59.1%), and GA data on all 208 (100%) participants. Direct before-and-after analysis was possible for the 117 respondents for whom complete and valid upfront surveys, follow-up surveys, and GA data were collected; this analysis answered whether engagement with SmartAPPetite was associated with changes in consumption.

### 3.1. Participant Characteristics

The median age of participants was 33; 66% were female. 69% of participants reported that they were already regular patrons at the farmers' market; the other 31% visited the market only infrequently, or for the first time the day they were recruited. Nearly 85% of participants had a household income of at least $50,000 per year, and over 20% had a household income of $100,000 or more. The group was very health conscious: 36% of participants were either very or extremely concerned with their health, while 44% reported above average or excellent health, only 11% reported below average or poor health. Still, 18% of participants were obese (BMI > 30), below the national average of 25% [61]. As well, only 10 to 16% of participants were concerned with issues such as diabetes, heart disease, high blood pressure, osteoporosis, and high cholesterol, compared to 48% who were not concerned with any of the above. This bias toward food literate and health conscious consumers likely influenced the results of this research.

### 3.2. Engagement with Messaging

Participants provided information in the upfront survey to guide the team's development of the food messaging chains and help participants reach their food goals. Many participants noted an inability to obtain the foods they wanted either due to limited selection (26%) or difficulty finding them in stores (26%), suggesting the importance of providing information on the availability of foods, while only 10% of the participants were vegan or vegetarian, 72% were interested in learning more about organic foods, and 37% and 29% were interested in gluten-free or wheat-free foods, respectively. Participants most often indicated a desire to consume more local (94%) and seasonal foods (82%), vegetables (76%), and fruits (67%). Most participants wanted to decrease the amount of processed foods (83%), sugar (78%), fat (61%), and salt (57%) in their diets.

Using the information gathered from the upfront surveys, a series of messages were sent via text message to participants.  Table 1 shows the various message categories according to the number of participants flagged to receive them, the total messages created for that category, and the messages actually sent. Sub-categories reflected a range of preferences for food goals, medical concerns, and specialty foods which, when checked by the participant, allocated importance to corresponding messages. Because many messages were aligned with multiple sub-categories (and thus were counted more than once), the total number of “messages sent” is higher than the total number of messages.

During the 8–10 week message deployment phase, GA reported a total of 30,605 messages sent to 208 participants, representing an average of about 15 messages per week per participant. GA was used to track visits to internal web pages and direct links to other websites subsequent to receiving text messages (Table 2). The most popular form of interaction consisted of visiting URLs that provided further healthy eating tips (82%). On average, participants viewed 13.5 tips each throughout the study period, or nearly 2 per week of participation.

Two-thirds of participants “checked in” to the farmers' market at some point during the study, and thus received additional market-specific messages. These two-part messages contrasted with typical daily messages by combining nutritional messaging (e.g., “Looking for a good source of protein, fibre and omega-3s?…”) with information about a vendor who sold relevant products to help the participant meet their dietary goals (“…Visit Kosuma upstairs for tasty, high-quality energy bars!”). On average, participants checked in 4.2 times, or once every 2-3 weeks, but a group of nearly half of the participants checked in to the market nearly every week. Some participants were highly active in visiting healthy eating tips and checking in to the market: over 20% of participants were checking in and “liking” tips multiple times per week.

Fewer participants used the “like” function (41%) or followed subsequent outbound links (33%). These values equate with 7.3 total likes per person and 2.5 visits to external websites from the messaging. As with participation in other aspects of the study, among those who did use the like function or visit outbound links, participation was high: some participants liked nearly every message sent and followed most outbound links.

The correlation between participation in one type of interaction with other types was assessed using Pearson product-moment correlation coefficients (*R* values). For instance, “liking” tips is strongly correlated with checking in to the market (*R* = 0.891), while following outbound links was less strongly correlated with checking in to the market (*R* = 0.315) and liking tips (*R* = 0.370). The weaker relationship between checking in and visiting outbound links may reflect a substitution effect, whereby participants who were unable to visit the market used the app as a way to obtain information on other healthy and local food.

**Figure 1:**
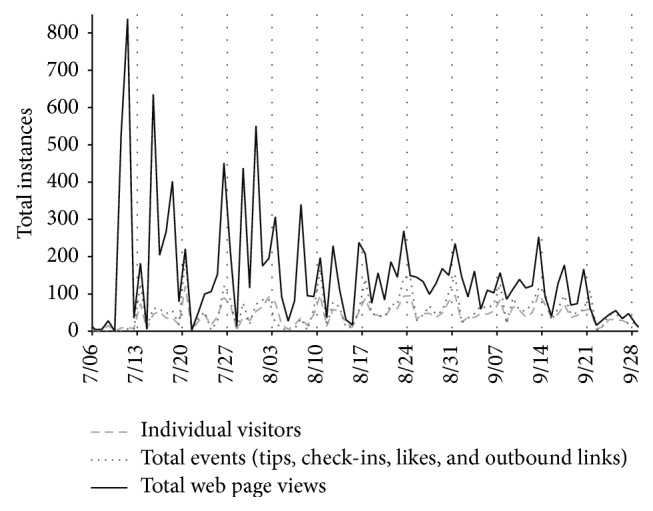
Daily URL visits to key components of the website.

An examination of daily URL activity across the study period is shown in Figure 1, including the number of individual visitors to the site, total events achieved (tips, check-ins, likes, and outbound links), and total web page views. As expected, participation on the website increased on Saturdays (market days) and on days corresponding with raffle drawings for market coupons and other special notifications. Spikes in the daily page views on the site earlier in the study period (>400 on 6 occasions) likely reflect that new participants visited the site more frequently to familiarize themselves with the content. Thereafter, participation was mainly focused on specific tips, recipes, and vendor spotlights.

### 3.3. Participant Reactions

At the end of the 8–10 week study period, participants were invited to complete a follow-up survey and an in-depth telephone interview. Analysis of the 123 follow-up surveys revealed that 80% of participants believed they had benefitted from the study in some way, while 46% believed the messaging had changed their food purchasing, eating habits, food knowledge, and/or health. The percentage of people very or extremely concerned with their health also increased from 34 to 47%. The percentage of participants who found the messaging very or extremely useful for various topics was highest for learning about seasonal (53%) and local (47%) foods, and lowest for topics such as produce storage/prep (32%), recipes (38%), and vendor sales (39%).

These levels of self-reported benefit and behavioural change were somewhat lower than those reported in previous studies using smartphone messaging apps [48, 62–64]. The self-reported high levels of food literacy and the generally healthy habits among participants may have contributed to the lower rates of satisfaction and behavioural change. Still, this information is valuable for improving various elements of SmartAPPetite for future intervention research.

Regarding direct suggestions to improve SmartAPPetite, 58% of participants wanted to see more messages about direct farmgate vendors, while only 29% wanted to see messages about grocery stores. Some users also wanted more real-time tailoring of messages (e.g., giving “thumbs up” or “thumbs down” to create a personalized message track), receiving more target messages early on market days, or receiving messages through another medium. Participants were most receptive to receiving future messages via e-mail, text message, or native apps (46%, 36%, and 33%, resp.), while fewer were interested in receiving messages through social networking websites.

### 3.4. Effecting Behaviour Change

One of the most noteworthy findings is that involvement with SmartAPPetite had a direct effect on consumption of healthy foods. Pearson's *R* correlations were calculated between the extent of participation in the app (measured by the number of visits, tips, likes, check-ins, and links visited) and changes in consumption of a range of foods (measured by self-report in the upfront and follow-up surveys). For the 117 users for whom complete upfront and follow-up survey results were available, the team found that while the app did not influence consumption behaviours across the board, greater participation with the app was strongly associated with improvements in healthy eating. These associations are shown in Table 3. Users who participated more with the app were more likely to see the following behavioural changes: decreased consumption of fruit juices, soft drinks, diet soft drinks, sugary foods, fast food, and prepared meals; and increased consumption of fruits, vegetables, and homemade meals. The users who saw the most positive changes in healthy behaviours had previously indicated their desire to eat less sugar and processed foods, and to receive tips about portion sizes. These users were also more likely to report that they found the app to be useful as a learning tool in every way surveyed (e.g., health benefits of specific foods, local foods, foods that are “in season,” sales by the market vendors, recipes, produce storage, and preparation suggestions).

## 4. Discussion

This paper evaluated the development and results of a smartphone intervention aimed at improving the knowledge, purchasing, and consumption of healthy, local food, based on validated theories of behaviour change and behavioural economic theory. Participation and satisfaction with the application was monitored qualitatively and quantitatively, including through interviews, surveys, and web analytics software. Results suggested that participants who engaged more actively with the application also experienced positive behavioural changes toward healthy eating (measured in increases in consumption of healthy foods and decreases in consumption of unhealthy foods), and were satisfied with the end result.

Although some participants did not engage closely with the app, the iterative development process created the opportunity to fix errors along the way as well as refine the application for future versions. Errors were seen in the message development process; some issues arose from glitches in the message deployment system, while others were reported by participants. Timelines for addressing issues were implemented depending on the severity and need of the error. For example, due to programming glitches, some messages were erroneously sent at odd hours (e.g., 1 a.m.), creating a considerable annoyance for many participants. These issues were typically unpredictable, one-time occasions, and the de-bugging process ultimately helped improve the utility of future messages.

While SmartAPPetite was successful in encouraging people to read “tips” and “check-in” to the market, considerably lower participation was seen with the “like” button. Participant feedback indicated that many people associated this option with the social networking site Facebook, and were reluctant to click it as they believed it would link SmartAPPetite to their Facebook account. This may have been due to a lack of communication at the outset regarding the benefits of clicking on the like button (e.g., providing additional tips on related topics) and the nonrelationship between the like button and that of Facebook.

Some participants were vocal about structuring delivery times so messaging did not arrive at inconvenient times, decreasing the volume of messages, spreading out the messages more evenly throughout the week, and improving the relevancy of messages to market-goers. The inconveniences led some participants to temporarily withdraw from the study, driving the research team to explore a more streamlined method for allowing participants to withdraw from, or rejoin, the study at will.

Some messaging was considered less effective at education or behaviour change. This is evident by the low percentage who found the recipes useful (38%). The current study was also unable to incorporate all allergies or intolerances in a responsive manner, and sometimes participants received messages which were inappropriate given their answers on the upfront survey. Future revisions will need to devise a more precise logic to screen messages and customize content based on each individual's upfront survey, ensuring a greater effectiveness in the next edition of SmartAPPetite.

Additionally, participants frequently self-reported high levels of food literacy during the interview process. Based on past literature citing a relationship among these factors, regular patronage of the farmers' market and various demographic characteristics may contribute to higher food literacy [65, 66]. This presented a substantial challenge in further educating participants about healthy eating and dietary changes. Since the intended sample for the regional SmartAPPetite project will range more greatly among the general public, however, this is only a minor concern.

Given lessons learned from this first study, various future steps must be taken to ensure the effectiveness of SmartAPPetite across a range of local food environments. Continued engagement with farmers and other local food vendors is necessary to expand SmartAPPetite to different locations. These include farmgate vendors, “u-pick” facilities, restaurants specializing in local food, community supported agriculture, and other farmers' markets. Ideally, local food throughout the entire region of Southwestern Ontario will be captured by the next phase of SmartAPPetite, before expanding outside the region. The logistical process of “scaling up” will be made easier by consulting the wide range of farmer's associations, economic development associations, and local food networks in Southwestern Ontario. Within the app, the use of GPS tracking and locational messaging will connect users to nearby vendors.

Attaining coverage of all local food vendors in the region is necessary to achieve a future goal of the research, which is to move beyond effecting behavioural change in participants and eventually increase profitability and job opportunities in the local food economy. The primary means of expanding SmartAPPetite to become an economic development tool will be to create an in-house website to host vendor information. While nutrition information messages can be easily stored in a static format, vendor messages and information will need to change seasonally and as new vendors join SmartAPPetite. Thus, the challenge will be to create a system whereby vendor information can be constantly updated by a self-sustaining content management system.

## 5. Conclusions

This paper presented the results of a multidimensional smartphone-based intervention to increase knowledge about and rates of healthy food consumption. Principles from Atkins and Michie's [55] framework for behavioural change were used to design a tool which would address various factors which inhibit healthy choices and thereby support the use of behavioural economics-driven interventions to address healthy eating. Evaluation tools included an upfront survey, study monitoring with web analytics software, experience sampling, and follow-up surveys and interviews, all of which made the SmartAPPetite project responsive to participant interests and desires around local food-based health promoting behaviours.

Because of the short time-frame and limited resources for this study, the team did not attempt to demonstrate long-term behavioural change in the study population. Moving forward, however, it will be necessary to determine whether SmartAPPetite achieved the ultimate goal of long-term improvements in food literacy, purchasing, consumption, and health. There is reason to be optimistic about such a behavioural economic approach, given these words by Gittelsohn and Lee [53]: “persuasive strategies that promote knowledge and attitudes, create structural change, and nudge individuals toward healthier choices can better address the multifactorial issues contributing to an unhealthy diet or food environment” (page 60).

The study achieved this goal through the creation of food information chains which guided users from healthy eating tips, to recipes incorporating these foods, and finally on to specific vendors, who sold these foods, making healthy food choices more visible and thus easier to make. This is illustrated through analysis of survey, interview, and website participation data showing that participants made use of SmartAPPetite and self-reported positive behavioural change. Over a longer time period, therefore, it should be possible to demonstrate whether the nudging of participants via the SmartAPPetite project has a positive effect on sustained behavioural change in healthy eating, local food purchasing, or health outcomes.

## Figures and Tables

**Table 1:** Message categories sent to participants.

Category	Subcategory	Number of participants marking category	Total messages created	Messages sent	% sent
Goals	Local foods	74	100	28	28.0%
	Seasonal produce	73	77	26	33.8%
	Processed food	69	102	37	36.3%
	Losing weight	60	74	35	47.3%
	Portion sizes	59	26	12	46.2%
	Sugar	40	19	6	31.6%
	Variety of foods	25	130	36	27.7%
	Fish	21	18	12	66.7%
	Salt	18	27	10	37.0%
	Vegetables	17	92	19	20.7%
	Fat	11	41	20	48.8%
	Fibre	10	49	25	51.0%
	Protein	8	22	6	27.3%
	Red meat	6	18	15	83.3%
	Fruits	4	60	17	28.3%
	Whole grains	4	23	13	56.5%
	Poultry	3	18	11	61.1%
	Nut-free	3	4	1	25.0%
	Gaining weight	3	3	1	33.3%
	Save money	2	22	7	31.8%
	Milk alt.	1	13	2	15.4%
	Milk and dairy	0	45	6	13.3%

Medical concerns	High blood pressure	1	94	49	52.1%
	High cholesterol	1	70	42	60.0%
	Heart disease	0	81	47	58.0%
	Diabetes	0	71	41	57.7%
	Osteoporosis	0	45	6	13.3%
	Lactose-free osteo	0	2	0	0.0%

Specialty foods	Organic foods	13	10	6	60.0%
	Vegetarian	10	85	34	40.0%
	Gluten-free	4	38	20	52.6%
	Vegan	1	34	12	35.3%
	Wheat-free	1	18	8	44.4%
	Lactose-free	0	22	9	40.9%
	Soy-free	0	11	4	36.4%

Other	Liver healthy	1			
	Special vendors/treats		37	5	13.5%

**Table 2:** Recorded “events” from Google analytics.

	Total recorded events	Participants in category	Average events per person	*N*	% using function
Followed links to tips	2313	171	13.5	208	82.2%
Checked in to market	583	139	4.2	208	66.8%
Liked tips	624	85	7.3	208	40.9%
Followed links to other websites	170	68	2.5	208	32.7%

**Table 3:** Pearson's *R* correlations between food consumption and level of engagement with SmartAPPetite.

	Visits	New visits	Tips	Likes	Check-ins	Links
Fruit juice	−0.30^*^	0.02	−0.30^*^	−0.26^*^	−0.35^*^	−0.07
Soft drinks	−0.23^*^	−0.06	−0.24^*^	−0.34^*^	−0.30^*^	0.01
Diet soft drinks	−0.12	0.03	−0.13	−0.16	−0.24^*^	−0.04
Caffeinated beverages	−0.09	0.01	−0.08	0.01	−0.04	−0.14
Fruit	0.01	0.06	0.02	0.10	0.03	−0.07
Vegetables	0.13	0.05	0.14	0.29^*^	0.23^*^	−0.08
Whole grains	0.00	−0.07	0.01	0.06	0.00	−0.10
Milk and dairy	−0.03	−0.18^*^	−0.03	0.04	−0.07	−0.11
Milk alternatives	−0.13	−0.03	−0.13	−0.12	−0.10	−0.09
Fish	−0.01	−0.04	−0.01	−0.01	−0.01	−0.04
Red meat	−0.04	−0.10	−0.03	0.02	−0.06	−0.08
Eggs	−0.06	−0.11	−0.06	0.02	−0.05	−0.08
Poultry	−0.05	−0.12	−0.05	−0.01	−0.05	−0.05
Sugary foods	−0.08	−0.31^*^	−0.08	−0.11	−0.13	−0.04
Fast food	−0.04	−0.14	−0.05	−0.08	−0.02	−0.07
Other restaurants	−0.03	0.02	−0.01	0.09	0.03	−0.05
Bakeries	−0.06	0.16	−0.05	−0.06	−0.01	−0.03
Prepared meals	−0.10	0.06	−0.10	−0.07	−0.07	−0.02
Homemade meals	0.06	0.01	0.07	0.23^*^	0.17	−0.03
BMI	0.01	0.10	0.01	0.00	0.04	−0.08

^*^Correlation is significant at the 0.05 level (2-tailed).
